# Leeds-Keio Ligament for the Reconstruction of Delayed Patella and Quadriceps Tendon Rupture: Time to Revisit?

**DOI:** 10.7759/cureus.82633

**Published:** 2025-04-20

**Authors:** Gopalkrishna G Verma, Jawad Sultan, Kan Nyunt, Mobeen Ismail

**Affiliations:** 1 Trauma and Orthopaedics, Manchester University NHS Foundation Trust, Manchester, GBR; 2 Trauma and Orthopaedics, Manchester Univeristy NHS Foundation Trust, Manchester, GBR

**Keywords:** elderly, knee extensor mechanism rupture, leeds-keio ligament, patella tendon rupture, quadriceps tendon rupture, synthetic graft, total knee arthroplasty

## Abstract

Ruptures of the knee extensor apparatus in elderly individuals or following total knee replacement present a significant functional limitation and surgical challenge. The Leeds-Keio ligament, a synthetic graft, has been historically used for reconstruction but has seen declining popularity with advancements in biological grafts. This study revisits the application of the Leeds-Keio ligament in managing chronic and complex extensor mechanism ruptures. It is a retrospective review of five patients (mean age 73.6 years) who underwent surgical reconstruction of the ruptured patella or quadriceps tendons with the Leeds-Keio ligament between 2022 and 2025. The surgical technique involved a figure-of-eight configuration to bridge or augment the repair. The postoperative protocol included initial immobilisation followed by a progressive rehabilitation program. All patients achieved satisfactory functional recovery with independent mobilisation at the latest follow-up, with some exhibiting mild residual extension lag. The diverse presentations included one case with a chronic 10 cm gap quadriceps tendon rupture that required quadriceps V-Y plasty and bridging with Leeds-Keio ligament. Three cases with patella tendon ruptures after total knee arthroplasty (TKA) had patella tendon reconstruction with the Leeds-Keio ligament, and notably, two TKA dislocations required revision to a hinged knee prosthesis for stability. Another case involved a re-ruptured primary patella tendon repair four weeks post surgery that needed reconstruction with a Leeds-Keio ligament. No major complications, such as deep infection or graft failure, were observed in this small series. The Leeds-Keio ligament may be a viable option for reconstructing complex chronic patella and quadriceps tendon ruptures in elderly patients, including revision scenarios and those with concomitant TKA complications, providing satisfactory early functional outcomes. Careful patient selection and individualised treatment strategies are essential to optimise outcomes.

## Introduction

The knee extensor apparatus is crucial for knee extension and endures substantial physiological loads of two to three and a half times body weight during daily routine activities [1]. In elderly individuals, rupture of the extensor mechanism can occur following traumatic falls onto a flexed knee or seemingly low-energy minor actions like rising from a chair. The resultant impairment of active knee extension, perceived instability, and reduced mobility due to the inability to lock the knee present significant functional limitations. The management of a missed chronic rupture or those occurring after total knee arthroplasty (TKA) poses serious and significant clinical challenges. Extensor apparatus rupture following TKA occurs in 0.17% to 2.5% of cases, with patella tendon involvement in 0.17% to 1% and quadriceps tendon involvement in 0.1% to 1.1% [2-6].

The diagnosis of an acute extensor mechanism rupture is typically straightforward in the presence of a clear history of traumatic knee injury, complete loss of active extension, and a palpable tendon gap. However, diagnosing atraumatic rupture causing insidious knee instability is more complex. Patients may notice the deficit only during exercise or when negotiating stairs. In these less obvious scenarios, clinicians should assess the knee for an extension lag under 20°, inability to fully extend from flexion, reduced extension strength, and compensatory hyperextension gait pattern to facilitate stable weight bearing [2, 7-8].

Sequential knee radiographs will demonstrate a high-riding patella as the quadriceps tendon retracts proximally after patella tendon rupture or a distally migrated patella in quadriceps tendon rupture. Although ultrasonography can provide initial confirmation of a tendon rupture, magnetic resonance imaging (MRI) is recommended for a comprehensive assessment, particularly in chronic cases. An MRI offers superior resolution and image quality, enabling detailed evaluation of tissue morphology, atrophy, intratendinous degenerations, precise measurements of tendon retraction and gap size, assessment of the surrounding muscle health (including fatty infiltration), and simultaneous evaluation of intra-articular knee structures to exclude associated injuries [9].

The Leeds-Keio ligament, a synthetic graft constructed from braided polyester, was previously utilised for the reconstruction of chronic patella and quadriceps tendon ruptures. It is designed to provide immediate mechanical stability, acting as a scaffold to facilitate eventual biological tissue ingrowth and integration. The ligament’s inherent strength and ease of handling made it a preferred choice for complex knee reconstructions in certain contexts [10]. Despite its initial success, its popularity waned with advancements in biological grafts and surgical methods. This article revisits the application of the Leeds-Keio ligament in the management of chronic and complex extensor mechanism ruptures in elderly patients, its early clinical outcomes, and explores its benefits and potential contemporary role in orthopaedic surgery.

## Case presentation

This retrospective review included a cohort of five patients (one male and four females) aged 65 to 81 years (mean age of 73.6 years) who underwent surgical reconstruction of the patella or quadriceps tendon rupture utilising the Leeds-Keio ligament. The study period encompassed a 3.2-year follow-up from 2022 to March 2025. Three patients presented with chronic ruptures (defined as greater than four weeks from injury) of the quadriceps or patella tendons, while two sustained acute patella tendon ruptures. All patients underwent preoperative MRI of the affected knee for a detailed evaluation of the rupture characteristics, including the location, extent of tendon retraction, and tissue quality, which will assist in preoperative surgical planning.

Surgical technique

The surgical approach and application of the Leeds-Keio ligament were tailored to the individual patient's specific clinical scenario and the nature of the tendon rupture. In all cases, the Leeds-Keio ligament was applied in a figure-of-eight configuration to either bridge the tendon gap or augment the primary repair.

*Patella* *Tendon* *Rupture*

For ruptures involving the patella tendon, the Leeds-Keio ligament was anchored between the tibial tuberosity and the patella. This typically involved the creation of bone tunnels in the distal pole of the patella and the tibial tuberosity. The ligament was then passed through these tunnels in a figure-of-eight fashion and secured under tension using either interference screws or non-absorbable sutures tied over a bone bridge or suture anchors. When a tunnel could not be created into the patella, for example, when associated with TKA and patella replacement, then the Leeds-Keio ligament was passed through the substance of the quadriceps tendon in the suprapatellar region.

*Quadriceps* *Tendon* *Rupture*

In the case of chronic quadriceps tendon rupture, the Leeds-Keio ligament was interwoven through the distal aspect of the quadriceps tendon and secured with sutures. Then it was passed through a bone tunnel created in the superior pole of the patella in a figure-of-eight pattern, providing robust distal traction and reinforcement. If the gap size was large, then a quadriceps V-Y plasty was initially performed to advance the tendon distally and reduce the retraction.

Patella Tendon Rupture With TKA Dislocation

**Figure 1:**
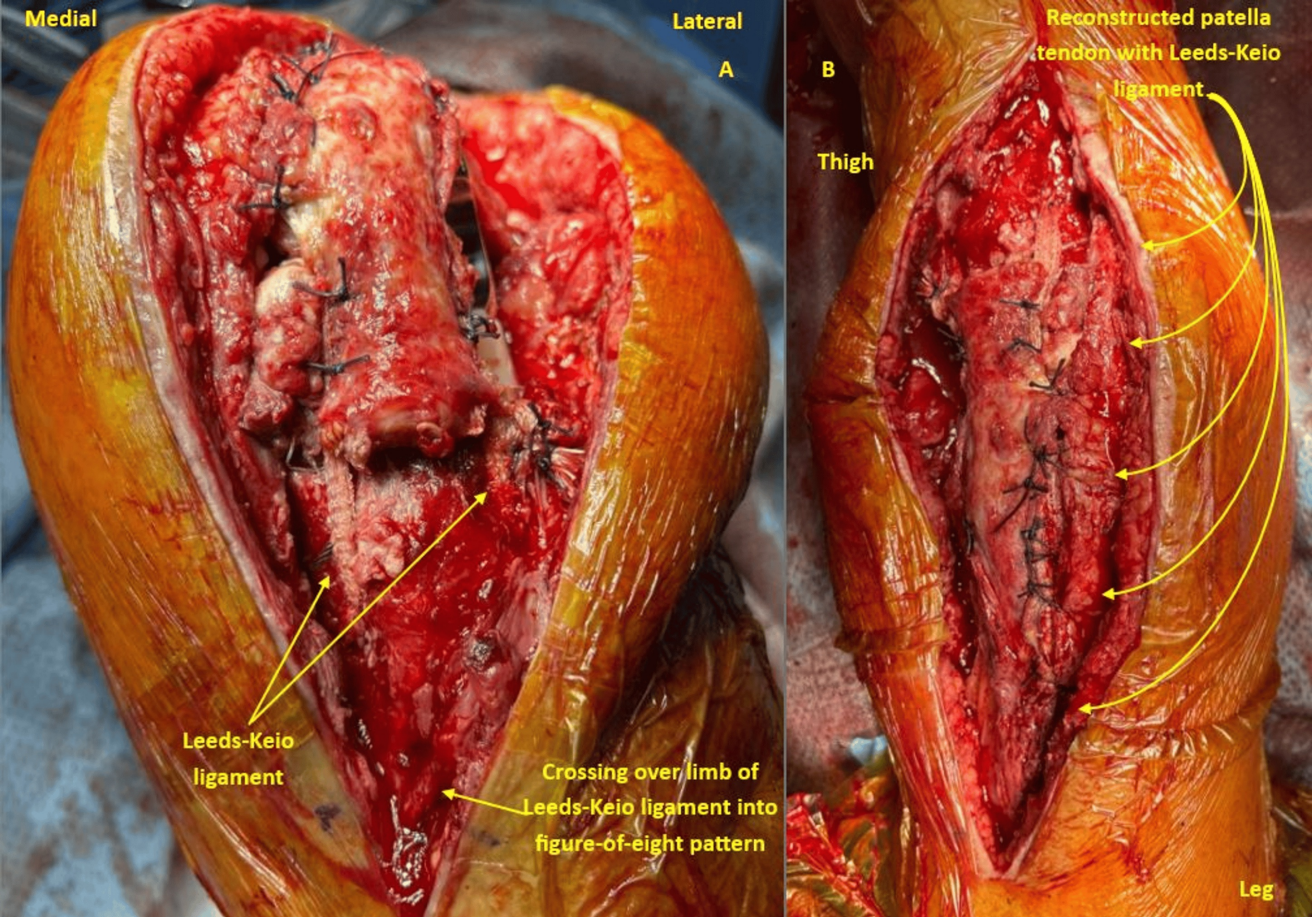
Intraoperative photographs of Patient 3 A: The Leeds-Keio ligament in a figure-of-eight configuration was passed proximally through the substance of the quadriceps tendon and distally secured through a bone tunnel in the proximal tibia. B: Patella tendon stumps were sutured to the Leeds-Keio ligament, and reconstruction was completed by covering it with retinaculum.

**Figure 2:**
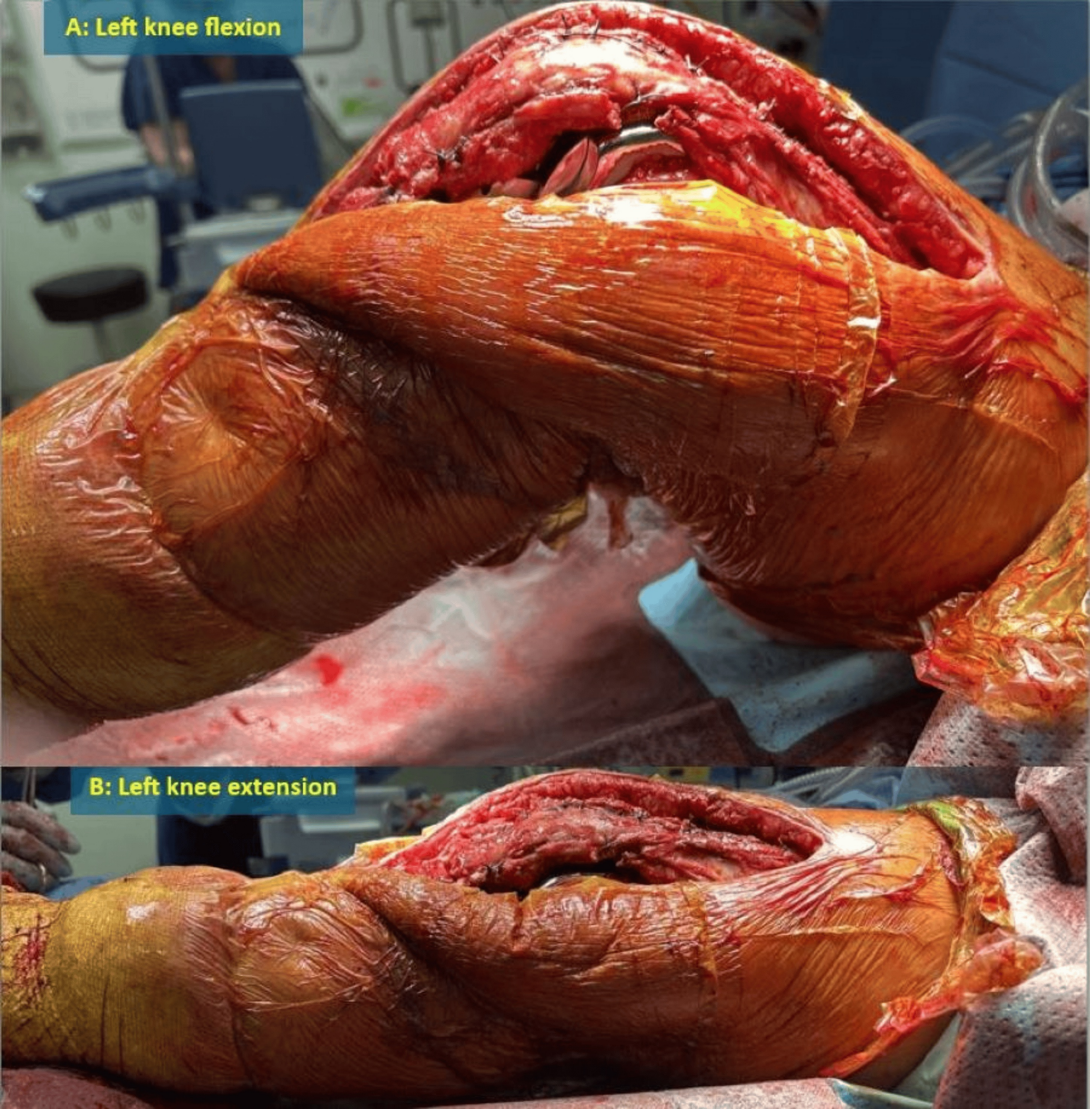
Intraoperative photographs of Patient 3 Left knee flexion (A) and extension (B) on the operation table after reconstruction of patella tendon rupture with Leeds-Keio ligament and revision of total knee arthroplasty to a total hinged knee arthroplasty

In these cases, TKA was revised to a hinged prosthesis if required to address the instability. The patella tendon was reconstructed using the Leeds-Keio ligament, woven in a figure-of-eight pattern between the proximal tibia and passed through the quadriceps tendon, secured with a laterally placed knot covered with soft tissue (Figures 1, 2).

Postoperative protocol

Postoperatively, all patients were immobilised in a hinged knee brace locked in full extension for the initial four weeks. Thereafter, the knee motion was progressively increased by 30 degrees of flexion every two weeks. Partial weight-bearing with the assistance of crutches was permitted for six weeks. A dedicated physiotherapist initiated an immediate postoperative rehabilitation program focusing on static quadriceps activation and straight leg raises. Prophylactic low molecular weight heparin was administered for six weeks to prevent venous thromboembolism until full weight-bearing was permitted.

Clinical data and outcomes

The patients' clinical data and outcomes are summarised in Table 1.

**Table 1:** Patient cohort details and outcomes yrs: years; M: male; F: female, QT: quadriceps tendon; PT: patella tendon; TKA: total knee arthroplasty

	Age	Tendon rupture	Surgery	Follow up (months)	Mean residual extension lag (degrees)	Mean flexion range (degrees)	Complications
1	65 yrs, M	Left chronic QT rupture with 10cm gap	Quadriceps V-Y plasty reduced the 6 cm gap, and Leeds-Keio ligament was used to bridge the 4 cm gap and augmented with a decellularised dermal patch	19	10⁰	120⁰	None
2	81 yrs, F	Left chronic PT re-rupture in a previously repaired three months earlier, PT rupture augmentation with fibre tape following TKA	Reconstruction with the Leeds-Keio ligament	36	0⁰	90⁰	None
3	75 yrs, F	Left PT rupture with TKA dislocation, four weeks post second stage TKA for previous infection	Reconstruction with the Leeds-Keio ligament with revision TKA	2.5	10⁰	90⁰	None
4	73 yrs, F	Left PT rupture with recurrent TKA dislocation	Reconstruction with the Leeds-Keio ligament with revision TKA	28	3⁰	90⁰	None
5	72 yrs, F	Right PT rupture in a previously repaired PT rupture with reinforcement with fibre tape four weeks ago	Reconstruction with the Leeds-Keio ligament	9	10⁰	90⁰	None

*Patient* *1*

**Figure 3:**
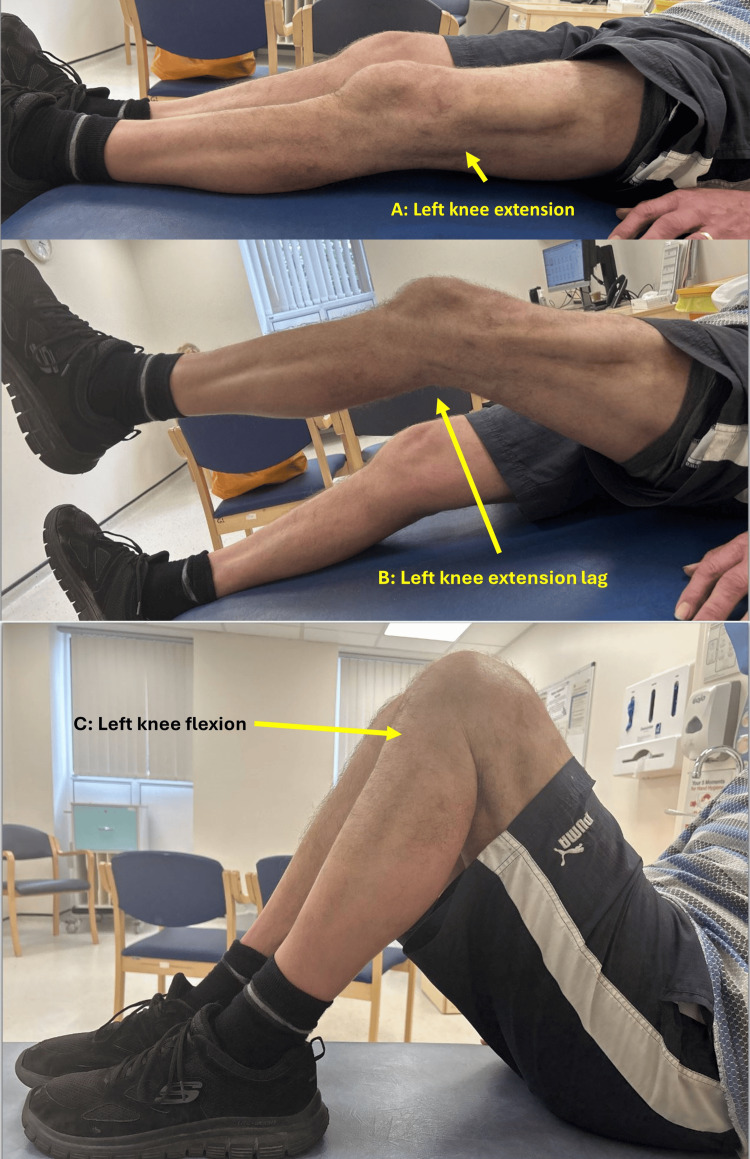
Patient 1's left knee range of movements A: left knee extension; B: left knee extension lag; C: left knee flexion

In October 2022, a 65-year-old male patient presented with a five-month history of left knee instability following a fall down the stairs. He used one stick to walk. His clinical examination revealed a left quadriceps tendon rupture with a substantial gap, indicative of chronicity. An MRI confirmed the diagnosis, showing a 10 cm proximal retraction of the quadriceps tendon. Due to a multitude of patient, administrative, and logistical factors, surgery was delayed by three and a half months. During surgery, the surgeon performed a quadriceps V-Y plasty to advance the tendon, which reduced the tendon gap by 6 cm. The Leeds-Keio ligament was used in a figure-of-eight pattern through the patella and quadriceps tendon to bridge the remaining 4 cm gap. This was further augmented with a decellularised dermal patch. At the most recent outpatient review, he walked independently without any walking aids (Figure 3).

*Patient* *2*

An 81-year-old female patient underwent a left TKA in October 2021. Postoperatively, she fell and ruptured her left patella tendon, which was reconstructed using a hamstring tendon graft augmented with fibre tape. Three months later, at a routine follow-up, she presented with an inability to extend her leg and left knee instability that had developed over the preceding weeks, with no reported trauma. Her clinical examination revealed a 20-degree left knee extension lag, and her patella was displaced superiorly. The MRI confirmed a rupture of the reconstructed patellar tendon. She underwent revision patella tendon reconstruction with a Leeds-Keio ligament in January 2022. At her most recent follow-up, she was able to mobilise independently without the need for any walking aids.

*Patient* *3*

The patient was a 75-year-old female who sustained a patella tendon rupture and TKA dislocation while rising from a chair four weeks post two-stage revision TKA. Following revision of the femoral component to a hinged knee and patella tendon reconstruction with a Leeds-Keio ligament, she mobilises independently with a single stick.

*Patient* *4*

A 73-year-old female patient with a recurrent TKA dislocation and patella tendon rupture underwent a primary patella tendon repair reinforced with Leeds-Keio ligament and revision TKA. At the latest follow-up, she reported being very pleased with the outcome and was independently managing all daily activities without knee-related issues.

*Patient* *5*

A 72-year-old female patient with a history of fragility fractures fell and avulsed her right patella tendon from the tibial tuberosity. This was repaired and reattached. Further reinforced with trans-osseous fibre tape and a FiberTak anchor (Arthrex Inc., Naples, FL). However, four weeks into her rehabilitation, she developed right knee pain during physiotherapy. Her imaging (ultrasound and MRI) revealed a re-rupture of the patella tendon. Consequently, she underwent revision patella tendon repair with augmentation using a Leeds-Keio ligament. She is mobilising independently without assistance and reported satisfaction with the outcome. She is being treated for osteoporosis.

## Discussion

Elderly individuals presenting with chronic ruptures of the patella or quadriceps tendon represent a significant clinical dilemma. This is further complicated when a previous patella tendon repair has failed in the setting of TKA. Direct repair is often unachievable due to tendon retraction and tissue degeneration, characterised by atrophic, friable tissue of poor quality. In these scenarios, reconstruction using various graft options aims to restore the extensor mechanism and knee function and improve patient outcomes. The choice of a graft, whether autograft, allograft, or synthetic, is determined by the extent of tissue damage, the presence of scar tissue, and the overall condition of the knee. Careful considerations for material properties, anticipated surgical outcomes, and the potential risk of complications like infection and graft failure are crucial [2-4, 6-7, 11].

The Leeds-Keio ligament is a synthetic braided polyester ligament engineered to serve as a scaffold to facilitate tissue ingrowth and eventual ligamentization. It provides immediate mechanical stability and possesses high initial tensile strength. It is readily available in various sizes, eliminating donor site morbidity and the risk of allograft-associated disease transmission. However, unlike autografts, this synthetic material does not undergo the same biological incorporation and remodelling, raising potential concerns regarding long-term durability, wear, and fatigue failure [12]. Additionally, there is a recognised risk of foreign body reaction from potential particle debris causing synovitis [13]. Furthermore, its biomechanical profile differs from natural tissue, lacking the complex hierarchical collagen structure, elasticity, and viscoelastic properties inherent in native ligaments and tendons [14]. While some studies have demonstrated acceptable long-term results with the Leeds-Keio ligament in anterior cruciate ligament reconstruction [15], specific data regarding its application in patella and quadriceps tendon reconstruction remains limited. Surgeons unfamiliar with its specific implantation techniques will require a period of adaptation and will need to overcome a learning curve.

Autologous hamstring tendon grafts are biological options that undergo biological incorporation and remodelling, potentially leading to a living graft that adapts to the joint environment. This eliminates the risks of disease transmission and immunological reactions. However, harvesting these grafts can result in donor site morbidity, such as hamstring weakness or pain during knee flexion. The variable size, strength, and quality of hamstring tendons, particularly in elderly patients, may limit their use in reconstructing large chronic tendon tears. The graft harvesting procedure adds slightly to the overall surgical time. Despite these drawbacks, hamstring autografts have demonstrated favourable clinical outcomes in various ligament reconstructions, including patella and quadriceps tendon ruptures [16].

Allografts, sourced from deceased donors, prevent donor site morbidity and provide readily available grafts in various sizes and types suitable for longer and larger defects or complex reconstructions. They can be advantageous when autograft tissue is insufficient or unsuitable. Allografts possess considerable mechanical strength, providing a fibrous framework for host tissue colonisation. While the risk of disease transmission and immune reaction is low with modern processing techniques, it is not eliminated, and the rate of biological integration is typically slower compared to autografts. Sterilisation can also weaken mechanical properties, and allografts may have higher failure rates [17].

This case series demonstrates the diverse clinical scenarios leading to rupture of the extensor apparatus in the elderly, including high-energy traumatic falls down the stairs on a flexed knee causing quadriceps tendon rupture, falls in the context of a TKA with patella tendon rupture and subsequent TKA dislocation, as well as lower-energy, seemingly trivial events like rising from a chair or during knee physiotherapy of the knee resulting in patella tendon rupture [2, 7]. This signifies the immense indirect forces that are transmitted through the knee extensor mechanism during daily activities [1]. While delayed presentation leading to chronicity presents inherent treatment complexities, the overriding objective remains restoration of the knee function and prevention of long-term disability. In this series, the Leeds-Keio ligament was used in various formats to augment repairs, provide structural support, and bridge the gaps in the retracted tendons of the patella or quadriceps, tailored to the individual case-specific clinical needs of each patient. Patients generally reported satisfactory functional recovery, with some exhibiting mild residual knee extension lag, a finding consistent across other studies in this field [2-8, 10-11, 14, 18-20]. Although this series is small, it contributes findings that align with other similar studies with small patient cohorts in the literature assessing the use of the Leeds-Keio ligament for reconstructing ruptured extensor apparatus tendons (Table 2).

**Table 2:** A review of previously published studies LK ligament: Leeds-Keio ligament; QT: quadriceps rupture; PT: patella tendon rupture; PF: patella fracture

Author	Year	Number of patients	Ligament	Location	Follow up (months)	Mean residual extension lag (degrees)	Mean flexion range (degrees)	Complications
Rand et al. [5]	1989	7	LK ligament	PT	24 (30-48)	20⁰	91⁰	None
Fujikawa et al. [14]	1994	1	LK ligament	PT	42	5⁰	87⁰	None
Aracil et al. [18]	1999	5	LK ligament	4 PT, 1 QT	56 (38–84)	5⁰-10⁰	98⁰ (90⁰-110⁰)	One superficial infection
Fukuta et al. [19]	2004	2	LK ligament	PT	38 (36–40)	5⁰	107⁰ (105⁰-110⁰)	None
Dobbs et al. [3]	2005	5	LK ligament	QT	2–36	6.6⁰	125⁰	Infection, re-rupture, and knee stiffness
Schoderbek et al. [6]	2006	6	LK ligament	QT, PT, PF	32 (12–60)	20⁰	90⁰-120⁰	Infection, re-rupture
Rust et al. [10]	2008	1	LK lig	QT	24	10⁰	90⁰	None
Kailash et al. [20]	2011	24	LK ligament	PT, QT	20	6.6⁰	125⁰	Patella fracture, ectopic bone formation
Rosenberg [8]	2012	5	LK ligament	Extensor tendon	-	15⁰	95⁰	-
Nam et al. [7]	2014	5	LK ligament	QT	24 (12-36)	10⁰	90⁰	Infection
Papalia et al. [4]	2015	5	LK ligament	PT, QT	24	10⁰	90⁰	Infection
Bonnin et al. [2]	2016	5	LK ligament	QT	36 (6–60)	10⁰-30⁰	105⁰ (90⁰-120⁰)	Infection, re-rupture
Olivia et al. [11]	2021	2	LK ligament	QT	24 (12 – 36)	10⁰	90⁰-120⁰	Infection, re-rupture, and knee stiffness

The limitations of this study include small sample size, heterogeneity of patient presentation and injury mechanism, and relatively short follow-up duration of three years. Furthermore, the lack of a comparative group limits the ability to definitively assess the superiority or inferiority of the Leeds-Keio ligament compared to other graft options in these specific clinical scenarios.

In summary, the choice between different graft options for extensor apparatus rupture should be individualised based on the patient's specific characteristics, the surgeon's experience and familiarity with surgical technique, and the available resources. Larger defects may necessitate longer grafts, which could favour allografts or certain synthetic options. Each graft choice presents its own set of advantages and disadvantages that must be carefully weighed to optimise patient outcomes. Although the Leeds-Keio ligament has shown promising long-term outcomes in anterior cruciate ligament surgery [15], evidence regarding its use in patella and quadriceps tendon reconstruction, particularly for chronic ruptures in elderly patients, remains limited. Future research should focus on conducting high-quality, long-term studies specifically evaluating Leeds-Keio synthetic grafts in these challenging clinical scenarios to better define their role in the surgical armamentarium.

## Conclusions

The selection between the Leeds-Keio ligament and biological grafts for reconstruction of chronic patella or quadriceps tendon ruptures in elderly patients is a complex decision influenced by several patient-specific factors, such as age, activity level, and the overall clinical picture. While autografts and allografts are often preferred for active individuals due to their strong biological integration and potential for long-term durability, allografts or synthetic options like the Leeds-Keio ligament may be viable alternatives in specific situations, such as revision procedures, limited autograft availability, or in patients with lower activity demands. In elderly patients where the quality of autografts and soft tissue healing potential are reduced and early weight bearing is required, synthetic grafts like the Leeds-Keio ligament offer clear advantages. Careful patient selection and a comprehensive discussion of the potential risks and benefits are crucial when considering these alternative grafts. Ultimately, the optimal choice depends on the individual patient's needs, as each graft type has its unique set of advantages and limitations. These findings highlight the intricate nature of managing these complex conditions and the necessity of personalised treatment strategies.
